# Utilization of Lead Nitrate to Enhance the Impact of Hydroxamic Acids on the Hydrophobic Aggregation and Flotation Behavior of Cassiterite

**DOI:** 10.3390/molecules29153692

**Published:** 2024-08-04

**Authors:** Saizhen Jin, Xiaobo Liu, Yun Feng, Yanfei Chen, Mengtao Wang, Qingfei Xiao

**Affiliations:** 1 Faculty of Land Resource Engineering, Kunming University of Science and Technology, Kunming 650093, China; 20230042@kust.edu.cn (S.J.);; 2 School of Metallurgy and Environment, Central South University, Changsha 410083, China

**Keywords:** lead nitrate, hydrophobic aggregation, cassiterite, hydroxamic acids, flotation

## Abstract

Lead nitrate (LN) is frequently employed as an activator in the flotation of cassiterite using hydroxamic acids as the collectors. This study investigated the effect of LN on the hydrophobic aggregation of cassiterite when benzohydroxamic acid (BHA), hexyl hydroxamate (HHA), and octyl hydroxamate (OHA) were used as the collectors through micro-flotation, focused beam reflectance measurement (FBRM) and a particle video microscope (PVM), zeta potential, and the extended DLVO theory. Micro-flotation tests confirmed that LN activated the flotation of cassiterite using the hydroxamic acids as collectors. Focused beam reflectance measurement (FBRM) and a particle video microscope (PVM) were used to capture in situ data on the changes in size distribution and morphology of cassiterite aggregates during stirring. The FBRM and PVM image results indicated that the addition of LN could promote the formation of hydrophobic aggregates of fine cassiterite, when BHA or HHA was used as the collector, and reduce the dosage of OHA needed to induce the formation of hydrophobic aggregates of cassiterite. The extended DLVO theory interaction energies indicated that the presence of LN could decrease the electrostatic interaction energies (V_edl_) and increase the hydrophobic interaction energies (V_hy_) between cassiterite particles, resulting in the disappearance of the high energy barriers that existed between the particles in the absence of LN. Thus, cassiterite particles could aggregate in the presence of LN when BHA, HHA, or a low concentration of OHA was used as the collector.

## 1. Introduction

Tin is widely used in welding, electroplating, alloys, electronic devices, and catalysis industries owing to its good ductility, conductivity, plasticity, and corrosion resistance [1]. In China, most of tin is extracted from primary tin ores consisting of cassiterite (SnO_2_). Cassiterite is extremely brittle and easily over-crushed to form fine particles during crushing and grinding [2,3]. Efficient flotation recovery of fine mineral particles has been a major challenge given the low probability of collision between micro-fine particles and air bubbles [2]. Hydrophobic aggregation is an important method for the flotation recovery of fine mineral particles, which enhances the collision probability between mineral particles and air bubbles by increasing the particle size [4,5]. The key to the hydrophobic flocculation of fine mineral particles is to increase the hydrophobicity of the mineral’s surface.

Hydroxamic acids, particularly benzohydroxamic acid (BHA), salicylhydroxamic acid (SHA), and alkyl hydroxamic acids, have been widely used as collectors to enhance the surface hydrophobicity and flotation behavior of cassiterite [5,6,7]. However, when hydroxamic acids are employed, activator reagents are frequently required to improve the cassiterite flotation recovery [6,8,9]. Lead nitrate (Pb(NO_3_)_2_, LN) is a common activator reagent that can improve the flotation recovery of a variety of minerals, including scheelite [10], wolframite [11,12], ilmenite [13], stibnite [14], sphalerite [15,16], and cassiterite [6,17]. The activation mechanism of LN is that LN makes the collectors easy to adsorb on the mineral surface and increases the adsorption level of collectors [10,12,14].

In the flotation of coarse cassiterite using hydroxamates as collectors and LN as an activator, lead species can adsorb on the cassiterite’s surface in the form of Pb(OH)^+^, thereby increasing the number of active sites on the surface and enhancing the adsorption amount of SHA [17]. Tian [6] investigated the effect of Pb^2+^ ions on the flotation performance of cassiterite using BHA as collectors. The experimental and computational results consistently indicated that, firstly, the dissolved lead ions can adsorb on the hydroxylated cassiterite surface by coordinating with two O atoms of the two hydroxyl groups on the surface, and then BHA molecules can adsorb onto the adsorbed Pb ion as a five-membered chelating ring.

To sum up, the activation mechanism of lead ions in mineral flotation has been the subject of extensive research. However, no research has been conducted on the effect of lead ions on the hydrophobic aggregation behavior of fine minerals. The objective of this study was to examine the effect of Pb on the hydrophobic aggregation behavior of fine cassiterite particles, using hydroxamic acids as collectors. Focused beam reflectance measurement (FBRM) and particle video microscope (PVM) were used to monitor the real-time evolution of particle size distribution and to capture images of cassiterite flocs during flocculation. This allows for the observation of alterations in the size distribution of the aggregates.

## 2. Results

### 2.1. The Effect of Lead Nitrate on the Flotation Behavior of Fine Cassiterite

**Figure 1:**
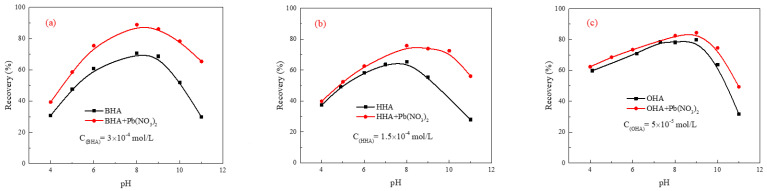
Recovery of fine cassiterite as a function of pH in the presence and absence of LN using (**a**) BHA, (**b**) HHA, and (**c**) OHA as collectors.

The flotation recovery of cassiterite as a function of pH in the presence and absence of LN when using BHA, HHA, and OHA as collectors is shown in Figure 1. As is shown in Figure 1, in the presence and absence of LN, cassiterite recovery increased with increasing the pH from 4 to 8.5, but decreased beyond this point. The flotation recovery of fine cassiterite in the presence of LN was higher than that in the absence of LN, indicating that the addition of LN could enhance the flotation recovery of fine cassiterite.

Moreover, there are great differences in the effects of LN on the flotation recovery of fine cassiterite using different types of collectors. As shown in Figure 1a, when BHA was used as the collector, the flotation recovery values of fine cassiterite after the addition of LN increased by 8.61%, 18.28%, and 35.51% at pH 4, 8, and 11, respectively. After the introduction of LN using HHA as the collector (Figure 1b), the flotation recovery values increased by 8.61%, 18.28%, and 35.51% at pH 4, 8, and 11, respectively. Figure 1c shows the effect of LN on the flotation recovery of fine cassiterite using OHA as the collector, and the flotation recovery values could increase by 2.54, 4.30, and 17.60 at pH 4, 8, and 11, respectively.

These results suggest that the activation effect of LN on BHA as a collector is the most effective, followed by HHA and OHA. When using BHA as the collector, the hydration layer on the cassiterite’s surface could prevent BHA from adsorbing directly on the cassiterite’s surface, but BHA could adsorb significantly on Pb-activated cassiterite’s surface. Therefore, lead ion could significantly activate the flotation of cassiterite using BHA as a collector [6]. When alkyl hydroxamic acids were the collectors, they adsorbed on the cassiterite’s surface through physical action, and the adsorption of hydroxamic acid increased with the length of the carbon chain [18,19]. Therefore, the effect of lead ion activation on HHA was greater than that on OHA.

### 2.2. The Effect of Lead Nitrate on the Hydrophobic Aggregation of Fine Cassiterite

The amount of hydroxamic acid required to float cassiterite reduced as the carbon chain length increased [18,19]. One benzene ring group corresponds to 3.5(–CH_2_–) in the linear alkyl group [20]. Therefore, BHA performed worse than HHA in the flotation recovery of cassiterite. The addition of Pb^2+^ can activate the flotation of cassiterite, reducing the required amount of collector for cassiterite flotation. However, no research has been conducted on how Pb^2+^ affects the hydrophobic aggregation behaviors of fine cassiterite utilizing BHA, HHA, and OHA as collectors. Therefore, the hydrophobic aggregation of fine cassiterite particles induced by BHA, HHA, and OHA in the presence of LN was investigated by FBRM and PVM.

#### 2.2.1. Effect of Lead Nitrate on the Hydrophobic Flocculation of Fine Cassiterite Using BHA

**Figure 2:**
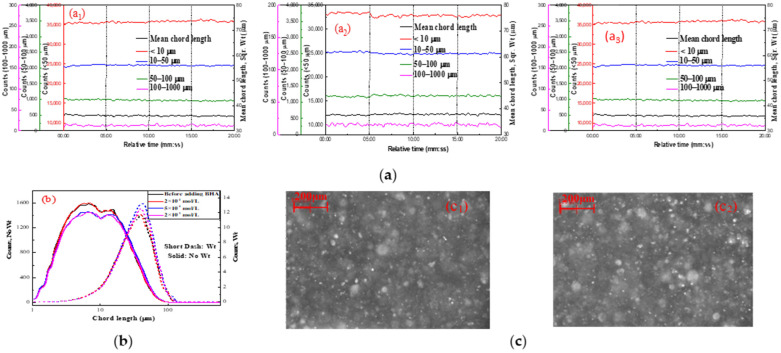
Aggregation of fine cassiterite in the presence of various BHA concentrations. (**a**) Counts and square-weighted mean chord length of cassiterite suspension as a function of time after adding (**a1**) 3 × 10^−4^ mol/L, (**a2**) 5 × 10^−4^ mol/L, and (**a3**) 2 × 10^−3^ mol/L of BHA; (**b**) non-weighted and square-weighted chord-length distributions of the cassiterite suspension before and after adding various concentrations of BHA at 20:00; (**c**) PVM images of cassiterite before (**c1**) and after (**c2**) adding 2 × 10^−3^ mol/L BHA at 20:00. (400 rpm; pH = 8.5~9.0).

The hydrophobic aggregation of fine cassiterite using 3 × 10^−4^, 5 × 10^−4^, and 2 × 10^−3^ mol/L of BHA as the collector in the absence LN was studied by FBRM and PVM, and the results are shown in Figure 2.

As illustrated in Figure 2a, the counts and square-weighted mean chord length of the cassiterite suspension remained unchanged with increasing the stirring time, indicating that the particle size distribution of cassiterite did not change after the addition of BHA. The chord-length distributions at the time point of 20:00, depicted in Figure 2b, show that the chord-length distributions of cassiterite particles after the addition of BHA remained the same as before. These results demonstrate that fine cassiterite could not form hydrophobic aggregates with the addition of BHA alone. Additionally, Figure 2c shows that no significant aggregates were observed in the PVM images following the introduction of BHA at 15:00, further demonstrating that aggregates did not form.

**Figure 3:**
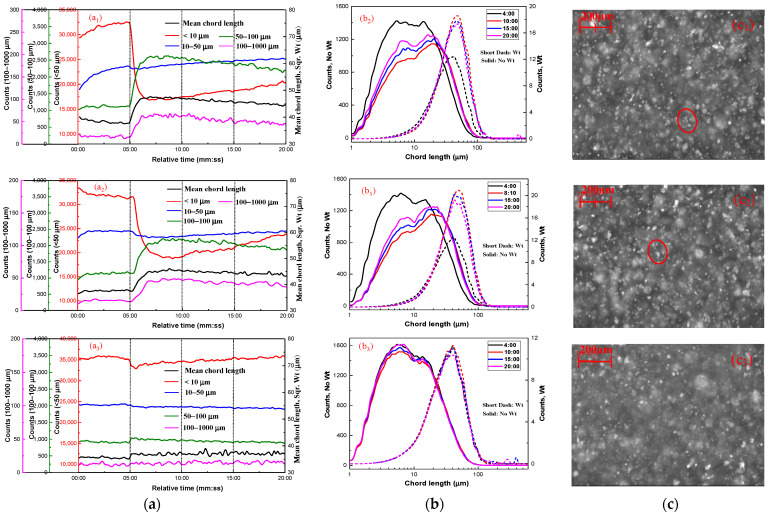
Aggregation of fine cassiterite in the presence of various BHA concentrations and Pb^2+^. (**a**) Counts and square-weighted mean chord length of cassiterite suspension as a function of time; (**b**) non-weighted and square-weighted chord-length distributions of cassiterite suspension at different times; (**c**) PVM images of cassiterite after adding BHA at 15:00. (C_(BHA)_ = 2 × 10^−4^ mol/L (**a1**,**b1**,**c1**), C_(BHA)_ = 5 × 10^−4^ mol/L (**a2**,**b2**,**c2**), C_(BHA)_ = 2 × 10^−3^ mol/L (**a3**,**b3**,**c3**); 400 rpm; pH = 8.5~9.0; C_(LN)_ = 6 × 10^−5^ mol/L).

Figure 3 shows the aggregation of fine cassiterite particles in the presence of 6 × 10^−5^ mol/L LN and various BHA concentrations. As shown in Figure 3(a1,b1) and Figure 3(a2,b2), with the increase in the relative stirring time in the presence of 3 × 10^−4^ mol/L and 5 × 10^−4^ mol/L BHA, the counts of −10 μm particles decreased and those in the 50–100 μm and 100–1000 μm ranges increased, along with an increase in the square-weighted mean chord length of the cassiterite suspension. The trends in the non-weighted chord-length distribution curves indicated a decrease in particle counts following the addition of LN and BHA. The peaks of the square-weighted chord-length distributions shifted from 39 μm at 04:00 to 46 μm at 15:00, indicating fine cassiterite particles aggregated after the addition of 3 × 10^−4^ or 5 × 10^−4^ mol/L BHA with LN. However, the counts of −10 μm particles slightly increased with an increase in stirring time after the time point of 10:00, suggesting that the aggregates dispersed during agitation. After the addition of LN and BHA, fine aggregates were observed in the PVM images at the time point of 15:00 (Figure 3(c1,c2)), further indicating that the same aggregates formed with the activation of LN.

When the dosage of BHA was increased to 2 × 10^−3^ mol/L (Figure 3(a3,b3)), the counts, square-weighted mean values, and distributions of chord length of the cassiterite suspension remained unchanged with the increase in the relative stirring time. This indicated that the cassiterite particles did not form hydrophobic aggregates, suggesting that a high dosage of BHA does not facilitate the formation of hydrophobic aggregates. Furthermore, no significant aggregates were observed in the PVM images at the 15:00 time point.

#### 2.2.2. Effect of Lead Nitrate on the Hydrophobic Flocculation of Cassiterite Using HHA

**Figure 4:**
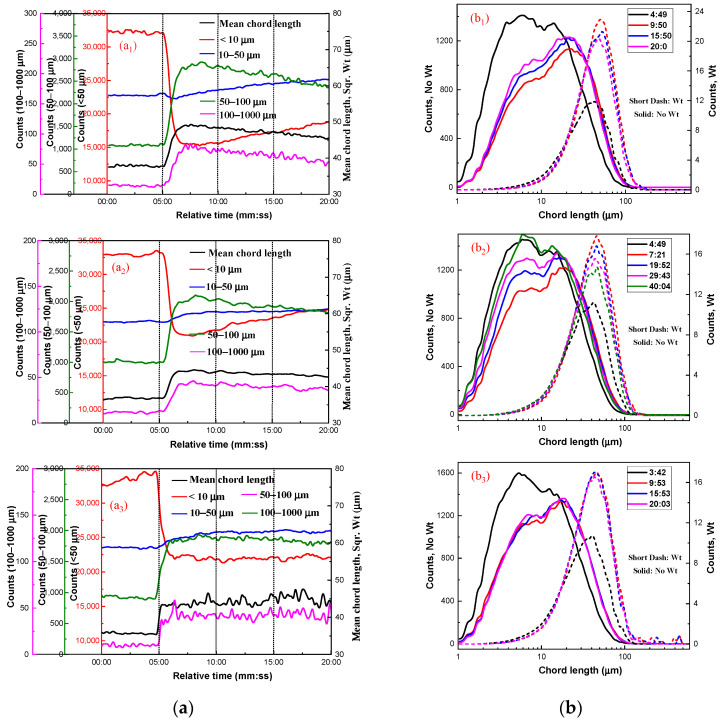
Aggregation of cassiterite under different HHA concentrations in the presence of LN. (**a**) Counts and square-weighted mean chord length of cassiterite suspension as a function of time. (**b**) Non-weighted and square-weighted CLDs of cassiterite suspension at different times. (C_(LN)_ = 6 × 10^−5^ mol/L; C_(HHA)_ = 5 × 10^−5^ mol/L (**a1**,**b1**), C_(HHA)_ = 1 × 10^−4^ mol/L (**a2**,**b2**), C_(HHA)_ = 4 × 10^−4^ mol/L (**a3**,**b3**); N = 400 rpm; pH = 8.5~9.0).

Fine cassiterite particles did not form hydrophobic aggregates when HHA was used as the collector [5], indicating that adding HHA alone did not result in hydrophobic aggregates of cassiterite. Figure 4 illustrates the effect of LN on the aggregation of cassiterite particles using 5 × 10^−5^, 1 × 10^−4^, or 4 × 10^−4^ mol/L HHA as the collector.

**Figure 5:**
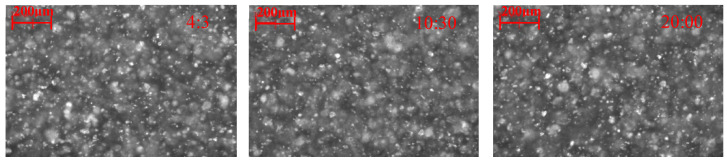
PVM images of cassiterite after adding 6 × 10^−5^ mol/L LN and 5 × 10^−5^ mol/L HHA at the time points of 4:30, 10:30, and 20:00.

As shown in Figure 4, the measured particle counts in each fraction and the chord-length distributions show that the hydrophobic aggregates of cassiterite particles form in the presence of HHA and 6 × 10^−5^ mol/L LN. However, the counts of −10 μm particles also slightly increased with the increase in the relative stirring time when the concentrations of HHA were 5 × 10^−5^ and 1 × 10^−4^ mol/L, indicating that the aggregates broke up during agitation. The particle counts in each fraction and the chord-length distributions remained unchanged when 2 × 10^−4^ mol/L HHA was added to the pulp, indicating that the strength of aggregates was improved and they did not break easily. The PVM images of cassiterite particles in the presence of LN and 5 × 10^−5^ mol/L HHA at the time points of 4:30, 10:30, and 20:00 are presented in Figure 5. In the images, fine aggregations can be observed, further indicating that the same aggregates formed with the activation of LN.

#### 2.2.3. Effect of Lead Nitrate on Hydrophobic Flocculation of Cassiterite Using OHA

**Figure 6:**
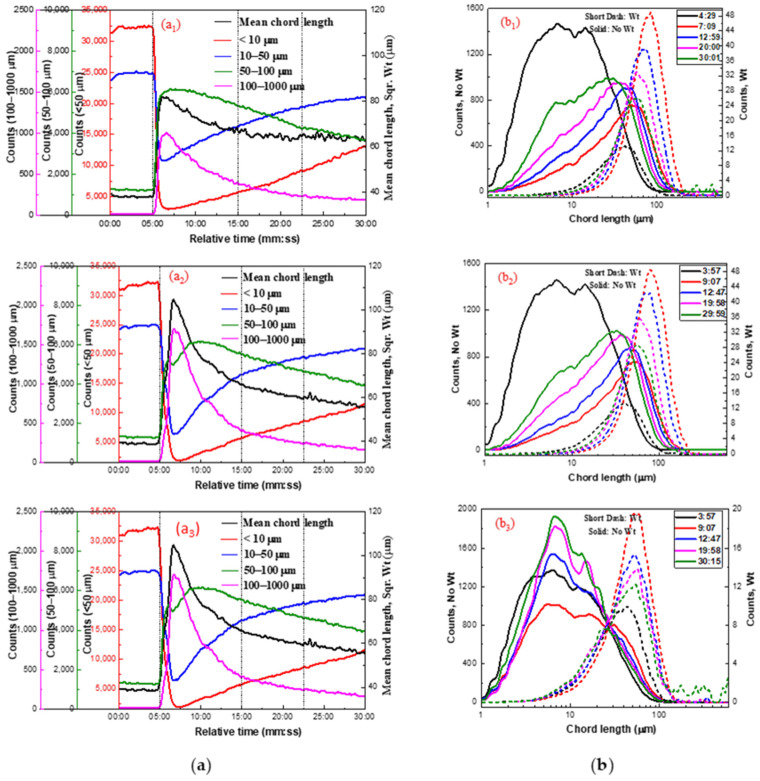
Aggregation of cassiterite by different OHA concentrations in the presence of LN. (C_(LN)_ = 6 × 10^−5^ mol/L; C_(OHA)_ = 5 × 10^−5^ mol/L (**a1**,**b1**), C_(OHA)_ = 1 × 10^−4^ mol/L (**a2**,**b2**), C_(OHA)_= 4 × 10^−4^ mol/L (**a3**,**b3**); N = 400 rpm; pH = 8.5~9.0).

**Figure 7:**
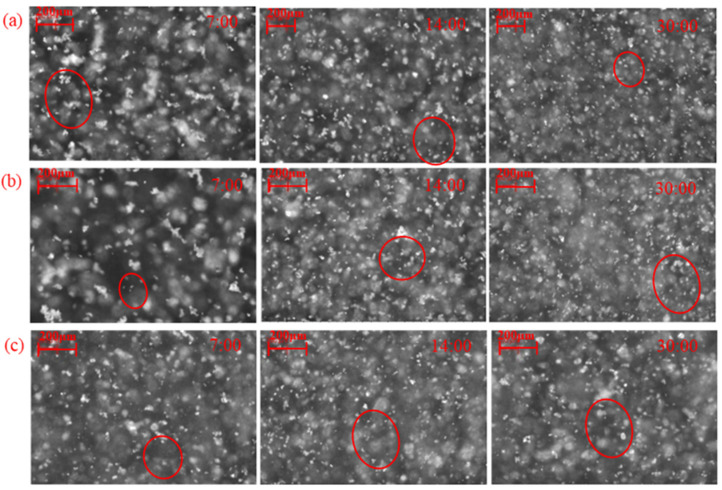
PVM images of cassiterite after adding OHA and LN. (C_(OHA)_ = 5 × 10^−5^ mol/L (**a**), C_(OHA)_ = 1 × 10^−4^ mol/L (**b**), C_(OHA)_ = 4 × 10^−4^ mol/L (**c**)).

According to previous research [5], in the absence of LN, the lowest concentration of OHA needed to induce the hydrophobic aggregation of fine cassiterite particles was about 1 × 10^−3^ mol/L. Figure 6 illustrates the aggregation of the cassiterite sample using 5 × 10^−5^, 1 × 10^−4^, or 4 × 10^−4^ mol/L OHA as the collector in the presence of LN. With the increase in the relative stirring time, the counts of −10 and 10–50 μm particles decreased, whereas the counts of 50–100 and 100–1000 μm particles increased, and the square-weighted mean chord length of the cassiterite suspension also increased. Moreover, the measured non-weighted chord-length distribution curves showed that the counts of particles decreased after the addition of LN and OHA. The peak of the square-weighted chord-length distributions shifted to the right after the addition of LN and OHA. This indicated that LN could promote the fine cassiterite particles to form hydrophobic aggregates when OHA was used as the collector. In the presence of LN, the addition of 5 × 10^−5^ mol/L OHA could result in the formation of hydrophobic aggregates of fine cassiterite particles, which could not in the absence of LN, indicating that LN could lower the concentration of OHA required to induce hydrophobic aggregates of fine cassiterite. The PVM images of cassiterite particles after adding OHA and LN are shown in Figure 7, showing larger aggregates at the 7:00 time point and distinct aggregates at the 20:00 time point, further indicating that hydrophobic aggregates formed with the activation of LN.

The PVM images depicted in Figure 7 clearly demonstrate that the particle sizes of the aggregates are significantly different using various dosages of OHA, which indicates that the dosage of OHA has an effect on the size of the generated aggregates. Additionally, at the same reagent dosage, the aggregate particle size reduced as the stirring time rose, suggesting that the aggregates may have broken down due to the stirring process. To more intuitively compare the aggregate sizes formed under various conditions and their size variations, the average sizes of cassiterite particles before adding hydroxamic acids (d1), at 9:00–10:00 (d2), and at 19:00–20:00 (d3) were calculated, and the results are listed in Table 1.

As shown in Table 1, d_1_ represents the initial particle size of the cassiterite, d_2_ represents the particle size after aggregation, and d_3_ represents the particle size after the fragmentation of the formed aggregates. The particle size of the aggregates induced by BHA and HHA was less than that of the aggregates induced by OHA. When the concentration of BHA or HHA was 3 × 10^−4^ or 5 × 10^−5^ mol/L, respectively, the particle size of the formed aggregates was larger than that of other concentrations. When 1 × 10^−4^ mol/L OHA was added, although the particle size of the aggregates formed at 10:00 was the largest (81.4 μm), with the continuous agitation of the pulp, the aggregates continued to break, and the particle size was only 61.4 μm at 20:00, which was smaller than the 64.9 μm particle size observed with the addition of 5 × 10^−5^ mol/L OHA. This indicated that the increase in the dosage of hydroxamic acid was also not conducive to the increase in the particle size of the aggregates. Furthermore, the results demonstrate that, in the presence of LN, OHA-induced aggregates are the largest and BHA-induced aggregates are the smallest, suggesting that, with the addition of LN, a longer carbon chain in the hydroxamic acid enhances aggregate formation.

According to Table 1, the aggregates induced by BHA and HHA have smaller particle sizes than those induced by OHA. When the concentration of BHA or HHA was 3 × 10^−4^ or 5 × 10^−5^ mol/L, respectively, the aggregate particle size was larger than at other concentrations. Although the particle size of the aggregates at 10:00 was the largest (81.4 μm) when 1 × 10^−4^ mol/L OHA was added, with the continuous agitation of the pulp, the aggregates continued to break, and the particle size was only 61.4 μm at 20:00, which was less than the 64.9 μm particle size observed with the addition of 5 × 10^−5^ mol/L OHA. This indicated that increasing the dosage of hydroxamic acid did not contribute to the increase in aggregate particle size. On the other hand, it was demonstrated that OHA-induced aggregates were the largest, while BHA-induced aggregates were the smallest. This result indicates that, in the presence of LN, an increase in the carbon chain length is also advantageous to the formation of aggregates.

### 2.3. Interaction Energy Estimation by the Extended DLVO Theory

**Figure 8:**
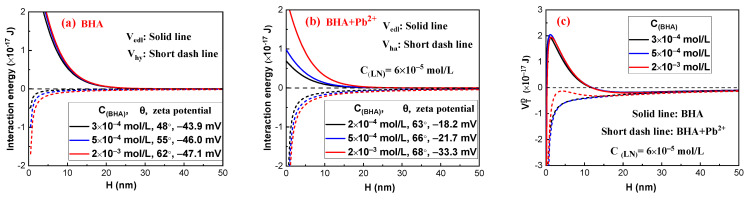
The EDLVO interaction energy diagram as a function of separation distance between cassiterite particles in the presence of BHA or BHA + Pb^2+^. (**a**) V_edl_ and V_hy_ in the absence of LN; (**b**) presence of _LN_; and (**c**) V_DT_ in the absence and presence of LN.

**Figure 9:**
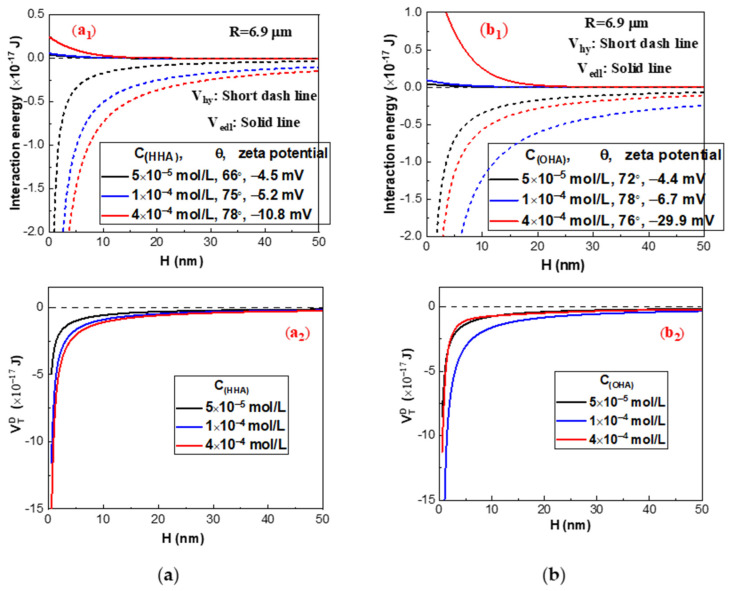
The EDLVO interaction energy diagram as a function of separation distance between cassiterite particles in the presence of LN and HHA (**a**) or OHA (**b**). HHA + LN (**a1**,**a2**) and OHA+LN (**b1**,**b2**).

The EDLVO theory is utilized for explaining the aggregation and dispersion of colloids as well as the interactions between particles in water [21]. In order to reveal the aggregation and dispersion behavior of cassiterite particles under different reagent conditions, the theoretical EDLVO interaction energies between particles were calculated using the measured contact angle and zeta potential. The results depicted in Figure 8 and Figure 9 were calculated using Equations (2)–(8) from the reference [5].

The electrostatic interaction energies (V_edl_) and hydrophobic interaction energies (V_hy_) between cassiterite particles at various dosages of BHA in the absence and presence of LN are in depicted Figure 8a,b, and the overall interaction energy (V_DT_) of van der Waals interaction energy (V_vdw_), V_edl_ and V_hy_, are presented in Figure 8c.

Figure 8c shows that high energy barriers exist amongst the cassiterite particles in the absence of LN. In order for mineral particles to form aggregates, the energy barrier between them must be broken by the input of energy through agitation. Yoon [22] found that the kinetic energies of coal samples with a particle size of 5 μm are 2.08 × 10^−19^ and 2.92 × 10^−20^ J at the mixing rates of 400 and 1000 rpm, respectively. The energy barriers amongst cassiterite particles were as high as 2 × 10^−17^ J. Therefore, the kinetic energy provided by mixing could not overcome such high energy barriers, and there were repulsive interactions between particles when they were in close proximity, resulting in the dispersion of particles.

As depicted in Figure 8a,b, the absolute value of the zeta potentials and contact angles increase with the BHA concentration, increasing the values of V_edl_ and V_hy_. In addition, the addition of LN could increase the contact angle of cassiterite and decrease the absolute value of the zeta potentials. Therefore, in the presence of LN, V_edl_ decreased while V_hy_ increased, resulting in the disappearance of the high energy barriers between particles in the absence of LN. Consequently, cassiterite particles could aggregate in the presence of various BHA concentrations when LN was applied. However, the energy barrier between particles disappeared at 2 × 10^−3^ mol/L BHA, but the attractive force energy between particles was low; as a result, the strength of aggregates formed between particles was very low, leading to aggregates that were easily broken by minor agitation.

Figure 9a,b depicts the EDLVO interaction energies between cassiterite particles when various concentrations of HHA or OHA were used as the collectors in the presence of LN. In Figure 9(a1), in the presence of HHA, the V_edl_ and V_hy_ values of cassiterite particles increase as the HHA concentration increases. V_edl_ in the presence of OHA in Figure 9 (b1) also increases as the OHA concentration increases with the increase in HHA concentration, while the greatest V_hy_ is observed in the presence of 1 × 10^−4^ mol/L OHA. V_DT_ in Figure 9(a2,b2) for cassiterite particles is a type of attraction energy, indicating that when two particles come together, they might be attracted to one another and form an aggregate. However, when 4 × 10^−4^ mol/L OHA was added, because the attractive force energy between particles was low, the aggregates unstably formed and could constantly break with agitation. Previous research demonstrated that in the absence of LN and only in the presence of various concentrations of HHA (5 × 10^−5^, 1 × 10^−4^, 2 × 10^−4^, 2 × 10^−3^ or 3 × 10^−3^ mol/L) or OHA (5 × 10^−5^, 1 × 10^−4^, and 4 × 10^−4^ mol/L), high energy barriers existed amongst cassiterite particles [5]. These results suggest that the addition of LN can eliminate the energy barrier between cassiterite particles and facilitate the formation of hydrophobic aggregates.

## 3. Material and Methods

### 3.1. Single Cassiterite Sample and Reagents

The cassiterite sample used in this study is identical to the one described in the previously published literature [5]. The D10, D50, D90, and volume-weighted mean particle size values were 2.08, 13.75, 36.04, and 16.72 μm, respectively. The hexyl hydroxamate (HHA) and octyl hydroxamate (OHA) utilized in this work are the same as those in [18]. Benzohydroxamic acid (BHA > 98.0%) was purchased from TCI Shanghai Chemical Industrial Development Co., LTD, Shanghai, China. Lead nitrate (LN, analytically pure) was used as the activator and purchased from Tianjin Kermil Chemical Reagents Development Centre. Note that lead nitrate is toxic and may lead to acute lead poisoning, so protective measures need to be taken during use. Hydrochloric acid (HCl) and sodium hydroxide (NaOH) were used as pH regulators and obtained from Sinopharm Chemical Reagent Co., Ltd., Shanghai, China. Deionized water (Resistivity = 18.2 MΩ·cm) was used throughout the experiments.

### 3.2. Micro-Flotation Tests

Micro-flotation tests were carried out using an inflatable hanging slot flotation apparatus (XFGC II) configured with a 40 mL cell. For each test, 2.0 g of cassiterite sample was first added to the cell along with a certain amount of distilled water. HCl or NaOH was then added to adjust the pulp pH. After adding 6 × 10^−5^ mol/L of lead nitrate (If necessary) and the desired dosage of the collector, the pulp was agitated for 3 min. A total of 1 × 10^−5^ mol/L MIBC was subsequently added and conditioned for 1 min. Flotation was performed for a total of 3 min.

### 3.3. Zeta Potential and Contact Angle Measurements

The zeta potentials of the cassiterite samples with the treatment of different reagents were measured using a Zeta PlusZeta potential analyzer (Brookhaven Instruments Corp., New York, NY, USA). With 0.005 mol/L KCl as the background electrolyte solution, the 30 mg single mineral was mixed into a suspension with a solid content of 0.05 wt% in 100 mL under the stirring of a magnetic agitator. The pH of the resulting suspension was adjusted to the desired value by HCl or NaOH, then desired glotation reagents were added in turn, and the action time of each reagent was the same as that of flotation. The contact angles of cassiterite were measured using a JY-82C video-based contact angle measuring device and the test procedure described in the literature.

### 3.4. Focused Beam Reflectance Measurement (FBRM) and Particle Video Microscope (PVM) Observation

The aggregation processes were detected in situ using a ParticleTrack G400 FBRM detector (Mettler Toledo, Zurich, Switzerland) and ParticleView V19 PVM (Mettler Toledo, Zurich, Switzerland). During agitation, FBRM and PVM were used to detect changes in particle size distributions and to observe the structures of particles and flocs. The sensors of the FBRM and PVM devices were submerged in the suspension during the measurement to synchronously obtain particle size distributions and images.

FBRM can detect particles between 0.5 and 2000 μm in size. During the measurement, the probe emits a focused laser beam that can rotate at the rate of 2 m/s to scan the particle that passes through the sapphire window. The “chord length (CL)” of the particle was calculated by multiplying its scanning time by its speed. Thousands of chord-length data were detected per second to produce the chord-length distribution (CLD). CL can represent the particle size, and the CL increases when flocculation occurs. And when the particle counts decrease in the suspension (flocculation occurs), the counts of the weighted chord length also decrease. The CLD of particles is a sensitive indicator that may represent the distribution of particle size. During measurements, CL data were collected every 10 s and PVM images were collected every 20 s. iC FBRM^TM^ software (version iC FBRMtm A4) was used to collect and analyze FBRM data.

Before measurements, the samples were ultrasonically dispersed for 5 min. A 300 mL slurry comprising 3.0 g of mineral sample was transferred into a 500 mL glass beaker for the measurements. At 400 r/min, the slurry was agitated with a magnetic stirrer [23]. After adjusting the pH of the slurry to 8.5–9.0, the FBRM detector and PVM commenced data collection. Five min later, the required amount of hydroxamic acid or hydroxamic acid with LN was added. Furthermore, the FBRM detector and PVM continued capturing data until the experiment concluded.

## 4. Conclusions

In this work, lead nitrate was introduced as an activator to enhance the impact of hydroxamic acids on the hydrophobic aggregation and flotation behavior of fine cassiterite. Based on the above experimental results and discussion, the following conclusions could be drawn:

(1) Micro-flotation tests suggested that the addition of LN could improve the flotation recovery of fine cassiterite and the activation effect of LN on BHA as a collector was the most effective, followed by HHA and OHA.
(2) FBRM and PVM results indicated that lead nitrate could facilitate the formation of hydrophobic aggregates of fine cassiterite using BHA and HHA as the collector, and reduce the necessary concentration of OHA to induce the formation of hydrophobic aggregates. In the presence of lead nitrate, when the amount of BHA was too large (2 × 10^−3^ mol/L), fine cassiterite particle could not form hydrophobic aggregates, and when the amount of OHA was too large (4 × 10^−4^ mol/L), the formed hydrophobic aggregates could be broken quickly.
(3) The EDLVO calculation results indicated that lead nitrate could decrease V_edl_ and increase V_hy_ between cassiterite particles, eliminating the high energy barriers that existed between the particles and favoring the hydrophobic aggregate using BHA, HHA, and a lower concentration of OHA as the collector.

This study did not extend the experiments to common gangue minerals. This is a significant aspect as the selectivity of hydrophobic flocculation is crucial for its practical applications in mineral processing. Future studies should investigate the behavior of common gangue minerals under similar conditions to better understand the selectivity and efficiency of hydrophobic flocculation.

## Figures and Tables

**Table 1:** Statistical results of aggregation formation and breakage rate.

Reagent	Concentration (mol/L)	d_1_ (μm)	d_2_ (μm)	d_3_ (μm)	R (μm/min)	s (μm/min)
BHA	3 × 10^−4^	38.3	47.1	44.5	1.76	0.26
	5 × 10^−4^	37.5	45.4	43.7	1.60	0.17
	2 × 10^−3^	35.6	37.1	37.2	0.30	0.01
HHA	5 × 10^−5^	37.8	48.4	45.6	2.12	0.28
	1 × 10^−4^	36.8	43.9	42.8	1.43	0.11
	4 × 10^−4^	35.7	44.4	44.4	1.75	0.002
OHA	5 × 10^−5^	37.2	74.7	64.9	7.30	0.98
	1 × 10^−4^	38.8	81.4	61.4	8.51	2.00
	4 × 10^−4^	37.4	48.5	44.8	2.23	0.37

## Data Availability

The original contributions presented in the study are included in the article material, further inquiries can be directed to the corresponding authors.
